# Land Premium Effects of Urban Rail Transit and the Associated Policy Insights for TOD: A Case of Ningbo, China

**DOI:** 10.1007/s40864-022-00180-z

**Published:** 2022-11-08

**Authors:** Xiongbin Lin, Buqing Niu, Wenting Liu, Jingjing Zhong, Qianqian Dou

**Affiliations:** 1 Department of Geography and Spatial Information Technology, Ningbo University, 818 Fenghua Road, Jiangbei District, Ningbo, Zhejiang Province China; 2 Ningbo Universities Collaborative Innovation Center for Land and Marine Spatial Utilization and Governance Research, Ningbo University, 818 Fenghua Road, Jiangbei District, Ningbo, Zhejiang Province China; 3 Donghai Academy, Ningbo University, 818 Fenghua Road, Jiangbei District, Ningbo, Zhejiang Province China

**Keywords:** Urban rail transit, Land premium effect, TOD, Land value capture

## Abstract

With considerable investments, mainly from local government budgets, the construction and operation of urban rail transit (URT) can exert significant spillover effects on the surrounding land use and land prices. In particular, China’s local governments are actively committed to developing their URT systems and promoting large-scale transit-oriented development (TOD) projects under the public land leasing policy. However, the connection between the land premium effects and TOD policy and practice is still lacking, particularly in the local government contexts, which exhibit significant policy and spatial heterogeneity. Thus, this research represents an attempt to better address this issue using the city of Ningbo as a case study. First, the premium effects of URT on land prices are examined, after which three crucial policy insights (land value capture [LVC], public–private cooperation [PPC], and urban regeneration) are proposed to enhance the effectiveness and efficiency of TOD, demonstrating its strong connection with the potential premium effects. The findings demonstrate that (1) local governments have adopted different innovative policies—with the ambition—to implement LVC; (2) assisted by PPC, the local rail transit authority can significantly amplify the premium effects, although it must still address the fair distribution of premiums across multiple stakeholders; and (3) transit-oriented urban regeneration can significantly influence land prices/land rents and subsequently generate significant gentrification, which will be further addressed by the TOD policy and practice.

## Introduction

As a major mode of large-scale urban public transportation, urban rail transit (URT) has been key to solving urban traffic congestion, as well as facilitating urban planning and development. By the end of 2020, 538 cities in 77 countries and regions globally had operational URT, with more than 33,000 km of operation and 34,000 stations [1]. In urban China, URT has facilitated significant development in recent decades, particularly under the National Transit Metropolis (*gongjiao dushi*) policies [2, 3]. Thus, at the end of 2021, a total of 50 cities in mainland China had operational URT systems, with a total of 283 lines and 9206 km of operation; the total annual passenger volume was as high as 23.69 billion [4]. The scale of China’s URT networks and passenger capacities continues to rank first globally, although this is influenced by the coronavirus disease 2019 (COVID-19) pandemic. Further, transit-oriented development (TOD) has become a popular strategy for executing local spatial planning and land development to cope with the fiscal burden occasioned by the rapid development of URT systems.

The planning, construction, and operation of URT can generate and exert significant spillover effects on the surrounding land use and prices owing to improved accessibility and agglomeration. For example, Knaap observed that land prices within 1 km of the rail station in Portland increased by 30–40% following the announcement of URT construction plans there [5]. Additionally, a meta-analysis revealed that URT can generate a significant increment in land prices in most cases [6]. The construction and operating costs of URT are enormous; thus, securing sustainable funding for URT certainly becomes a necessary responsibility of the local governments.

Following the policy of prioritizing investment in transport infrastructure, China’s URT has developed rapidly, extending gradually from large-scale to medium-sized cities. According to China’s 14th five-year plan, the operating mileage of URT is expected to reach 10,000 km by the end of 2025. Further, the *Opinions on Further Strengthening Urban Rail Transit Planning and Construction Management*, which is issued by the General Office of the State Council, state that the local governments that aim to build a URT system must satisfy several requirements regarding population scale, economic performance, and fiscal conditions. For example, their general public budget revenue and gross domestic product (GDP) must be over ¥30 billion and ¥300 billion, respectively. Additionally, the population of the urbanized area must be over three million. Since URT can generate value-added benefits on land prices, a potential strategy in TOD policy and practice involves determining how to better utilize the premium effect through effective land value capture (LVC) instruments, and this can help alleviate the fiscal pressure on the local governments.

Generally, the extant literature reports a variety of models for assessing the land premiums of URT, revealing the significant temporal and spatial heterogeneity of these externalities. Several studies have employed the concept and various LVC instruments to elucidate the government’s action toward dealing with the positive externalities. Indeed, many cities globally have adopted various LVC strategies to better finance URT as an additional revenue source. However, the externalities vary significantly across municipalities, and the implementation of LVC could be localized, particularly considering the differentiated policy and spatial development characteristics. Further, the land appreciation effect owing to URT cannot be fully exploited because of the complicated action mechanism as well as the policy environment. To implement LVC strategies, as well as to fully utilize the land premiums associated with local contexts, reasonable measures for dealing with these policy constraints or challenges must be effectively explored. Thus, this study is an attempt to elucidate the spillover effects of URT on land prices and the utilization of the externalities for increasing LVC under China’s public land leasing system and Ningbo city’s local initiatives. As an economic center of the Yangtze River Delta (YRD) of China, Ningbo is a coastal city in Zhejiang province, with a total population of 9.54 million and a GDP of ¥1459.49 billion by the end of 2021. Though there are dozens of cities with a URT system under operation in China, Ningbo is a typical case for understanding the land premiums driven by the URT as well as the associated insights for TOD planning and policy. Ningbo is a certificated transit metropolis issued by China’s Ministry of Transportation (MOT) in 2018. The Ningbo municipal government has issued a series of policies to promote TOD under the potential impacts of URT on surrounding land development and land prices. Thus, this study will better elucidate the spillover effect of URT on land prices in the local context, as well as the associated government’s local actions, thereby fully increasing LVC through local efforts and policy innovation.

## Literature Review

### TOD and Land Premium Effects

The TOD concept has emerged as a significant development strategy in the field of urban planning and transportation. Calthorpe was the first to clearly define the concept, stating its types, elements, and related principles [7]. In a broad sense, TOD refers to urban development activities that are guided by high-capacity rail transit infrastructure [8]. Boarnet and Crane defined TOD as the development of mixed and high-density land utilization near rail stations [9], particularly within a specific geographic area [10]. A previous study considered the concept, advantages, and principles of TOD, as well as the behavior of local governments toward ensuring the effectiveness of this new development mode. The continuous development of the research on TOD has yielded a diverse and complex theoretical, and policy and implementation framework involving transportation networks [11], an urban development model [12], and effective project financing and management [13]. Regarding China’s policy and planning efforts to build highly connected intracity or intercity rail transit systems, TOD has become a new perspective for reshaping the urban development pattern and land-use planning of urban China.

Regarding the potential for transportation and land-use integration under the TOD initiative, TOD has been widely applied in developed and developing countries. Considering the large-scale investment in the URT system, the adoption of a reasonable financing model is crucial for promoting the TOD policy and practice. URT can significantly boost land prices by improving transportation accessibility, as well as promoting population and economic agglomeration, thus optimizing land and economic development by increasing local vitality [14]. The extant literature reports diverse approaches for assessing the potential impact of multimodal rail transit, such as metro, heavy, and light rail, on land prices and rents. For example, Xu et al. observed that the subway in Wuhan, China, exerts a significant spillover premium effect on the surrounding land prices, with premiums of 16.7% and about 8.0% within 100 m and the following 100–400 m of the station, respectively [15]. Contrarily, several studies have revealed that rail transit could negatively affect the surrounding land prices because of the accompanying noise pollution and high crime rates around URT stations [5, 16, 17].

Indeed, the extant literature has shown the utilization of different models to comprehensively examine the effect of URT on land price increment. These models include hedonic pricing, multilevel logit, geographically weighted regression, spatial error, and difference-in-difference models [18–20]. In particular, considering the nonlinear and threshold effects of URT, new models based on big data and machine learning—such as gradient boosting decision trees—have been developed and applied [21, 22], producing mixed results regarding the impacts of URT on land prices. Such complex impacts might be due to contextual factors and methodological issues [6]. Thus, most studies have revealed the spatial–temporal heterogeneity of the impacts of URT on the surrounding land prices. For instance, URT exerts different degrees of impact on land prices at various stages of its development, ranging from planning and construction to operation [5, 23, 24]. Regarding the spatial issues, the land premium effects increase with the increasing proximity to the rail transit station (RTS) [25–27]. Additionally, the land premium effects would vary significantly across municipalities with differentiated policies and spatial development characteristics. In this sense, the impacts of URT on land prices or rents would be closely associated with local contexts—regarding the economic development and urbanization stage, motorization, rail transit structure, and traffic congestion conditions. Furthermore, considering the impacts of local contexts, additional studies are necessary to determine how local governments fully utilize such land appreciation effects under different spatial and policy conditions.

### Different LVC Strategies

LVC represents a concept for capturing additional land value from government actions and public investment. Considering the source of local government funds for URT investment and construction, LVC has also been widely employed in the URT sector. LVC is the process of recovering all or part of the land value appreciation via investments in URT [28], mainly to internalize the value-added benefits [29]. Currently, there are two mainstream LVC strategies: tax-based and development-based LVCs [20, 30–32]. The tax-based LVC instruments are generally implemented by the American-style public finance system [33]. The concept of land value taxation originated in 1879 when Henry George proposed the utilization of the land value appreciation of all residents for public facilities and services [33]. The development-based LVC is widely employed in the public-land-ownership system. “Rail + Property” joint development is one typical model of such an LVC instrument. For instance, Hong Kong has implemented the best practice of “Rail + Property” globally, with high efficiency and effectiveness to exploit land premiums to finance rail transit development. Specifically, the Hong Kong Government adopts the public–private cooperation (PPC) construction and development system by designating the lands around mass transit railway stations as comprehensive development zones. Thereafter, such lands are allocated to the Hong Kong Mass Transit Railway (HKMTR) corporation via a private agreement, and the latter selects the developers for joint development, following the existing market principles [35].

Owing to China’s public land leasing system, mainland Chinese cities can implement land concessions to achieve LVCs. However, there are some challenges regarding the sustainability of this value capture strategy [36]. On the one hand, the revenue from land concessions accrues on a one-time basis, when the construction of rail transit and the real estate market boom correlate highly. Conversely, the rail transit construction cycle is long, and the rail transit premium, which is recovered by the government, is generally not used as a source of funding for metro operations. Currently, the major cities in mainland China are gradually changing the value capture strategies of previous land sales and embracing the “Rail + Property” joint development model of Hong Kong to achieve the LVC policy goals. However, China still faces many challenges regarding the practice of such a “Rail + Property” joint development. For example, China’s land law stipulates that state-owned lands for business purposes must be publicly offered for sale through bidding, auction, or listing, and this presents strong constraints regarding the acquisition of lands along the rail transit route for local rail transit companies. Thus, the effective exploration of reasonable measures to mitigate these policy constraints or challenges is quite necessary for implementing LVC strategies, as well as fully utilizing the land premiums that are associated with local contexts.

## Research Design

**Fig. 1.**
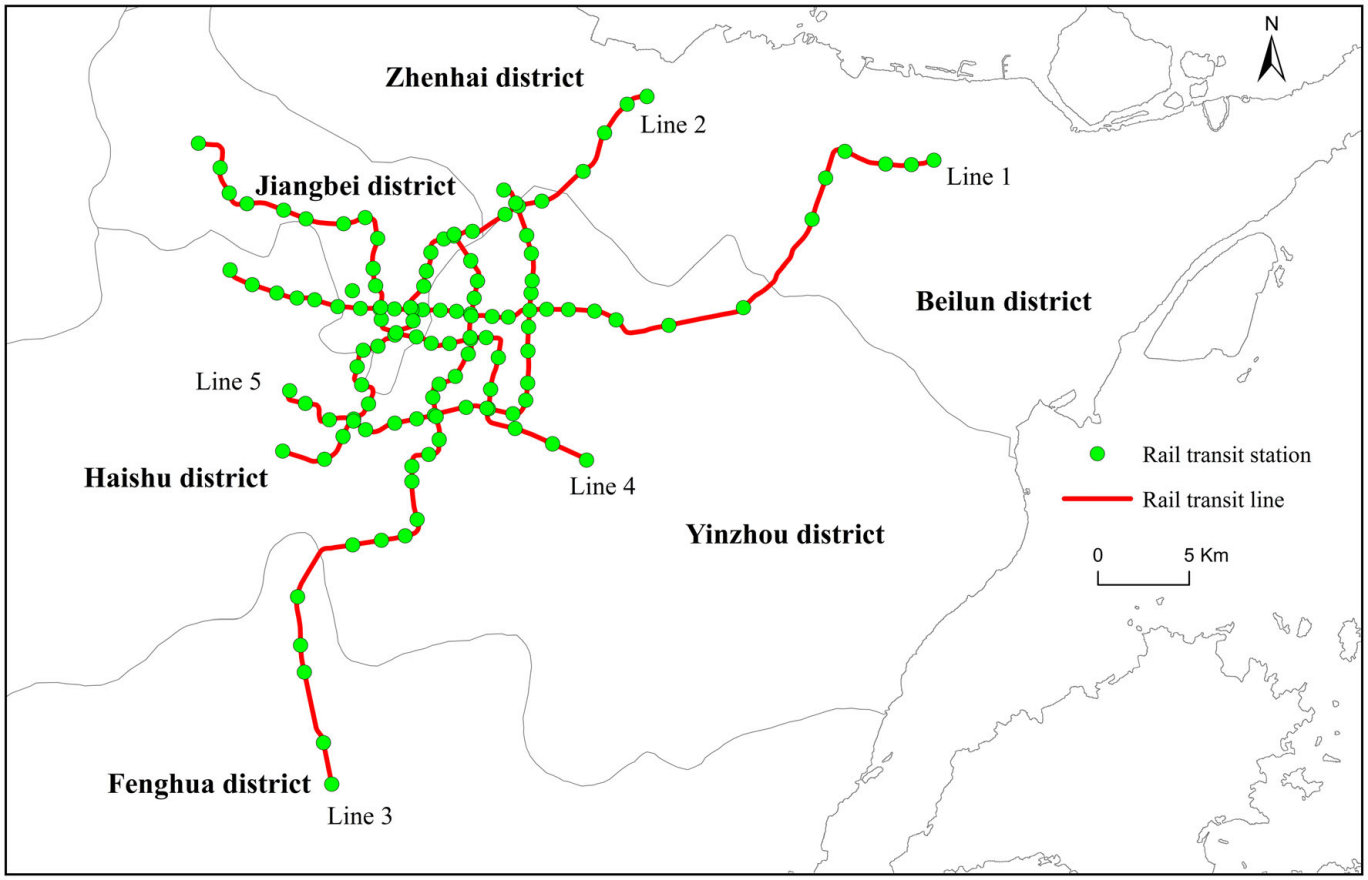
Operational URT system in Ningbo

Considering the rapid investment in and development of the URT system in urban China, examining its positive effects on the surrounding land prices, as well as its policy implications for rail transit investment and land-use planning, can further promote its effectiveness and that of TOD. Since the approval of the *Ningbo Rail Transit Network Plan* and *Ningbo Urban Rail Transit Construction Planning (2008–2015)*, Ningbo municipal government began the first round of its URT construction, opening the first rail transit line in 2014. After the approval of the *Ningbo Urban Rail Transit Construction Plan (2013–2020)* in 2013, the second round of construction commenced. By the end of 2021, the total mileage of the URT system in Ningbo reached 154.55 km, including five URT operational lines (Fig. 1). After the completion of the third phase of the Ningbo UTR construction plan, Ningbo will build a URT network with total mileage of over 270 km, which would inject new vitality into the economic and social development of the city.

**Fig. 2:**
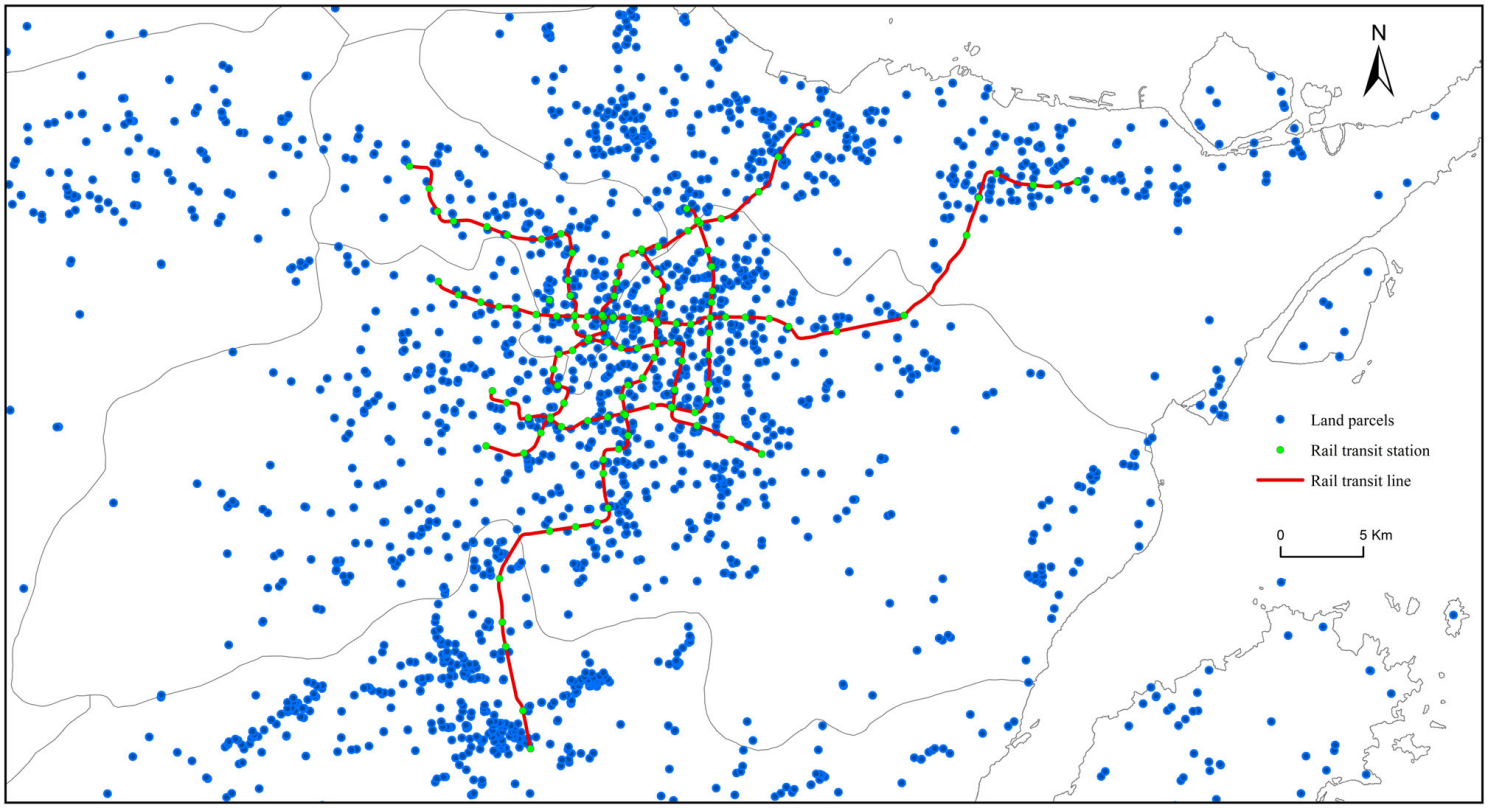
URT and land transaction record in Ningbo

Indeed, it is essential to elucidate and exploit the land premium effect, observing its implications on TOD based on PPC, LVC, and transit-oriented gentrification. To do this, the main objective of this study, which employed the city of Ningbo as a case study, is to first examine the land premium effect of URT on land prices by adopting the hedonic pricing models. For this study, a total of 5035 Ningbo land transaction data were collected between 2002 and 2019 (Fig. 2). The land transaction data were collected from the China Land Market Network (https://www.landchina.com/), which is governed by the Real Estate Registration Center of the Ministry of Natural Resources. These land transaction data contain information regarding the land-use type and land transaction dates, prices, and modes. Table 1 presents the basic information on the land transaction data and the other control variables. Second, after gaining a valuable understanding of the land premiums, this study proceeded to clarify three aspects of its policy insight for TOD, namely, LVC, PPC, and transit-oriented gentrification.

**Table 1:** Summary of the dependent and independent variables in the hedonic pricing model

Variable	Obs.	Mean	Std Dev	Min	Max
LTP (yuan/m^2^)	5035	2826.077	7837.914	0.300	119895.3
DisRTS (km)	5035	4.367	5.403	0.009	32.988
DisBTS (km)	5035	0.461	0.983	0.00018	15.017
DisPOI (km)	5035	0.258	0.393	0.00018	5.531
DisHSR (km)	5035	13.331	9.621	0.051	56.625
FAR	5035	1.317	1.122	0	8
RTO	5035	0.408	0.492	0	1

## Land Premium Effect of URT in Ningbo City

The hedonic pricing model is a popular method for examining the effect of URT on the increment of land prices. This model is widely employed in the real estate market, since the price of a building or land parcel would be determined by the internal factors (e.g., size, land-use type, or density) or the surrounding environment (e.g., distance to certain public services or urban center) [37, 38], but it is still necessary to understand such an impact by considering various independent variables under different local or regional contexts. The hedonic pricing model can be employed to examine the impact of each factor on the market prices of properties, such as buildings, neighborhoods, and location characteristics [18]. To elucidate the impacts of rail transit proximity on the surrounding land transaction prices (LTP), the existing literature [38] reported that the distances to the nearest rail transit station (DisRTS), nearest bus transit station (DisBTS), nearest public service point of interest (DisPOI), and nearest high-speed rail (DisHSR) stations were calculated in ArcGIS and applied to the hedonic pricing model (Table 1). The floor area ratio (FAR) was treated as a controlled internal variable of the land parcel. The first URT line in Ningbo became operational on May 30, 2014; some land parcels were transacted before this date, while others were transacted afterward. To better understand the impact of URT on land prices, a dummy variable was constructed to reflect the rail transit operation (RTO). RTO was assigned a value of 1 when the land transaction occurred after May 30, 2014; otherwise, the value was 0. Additionally, being the crucial external factors, the DisBTS, DisPOI, and DisHSR variables were implemented to further clarify the impact of the proximity of rail transit to the surrounding lands on LTPs.

**Fig. 3:**
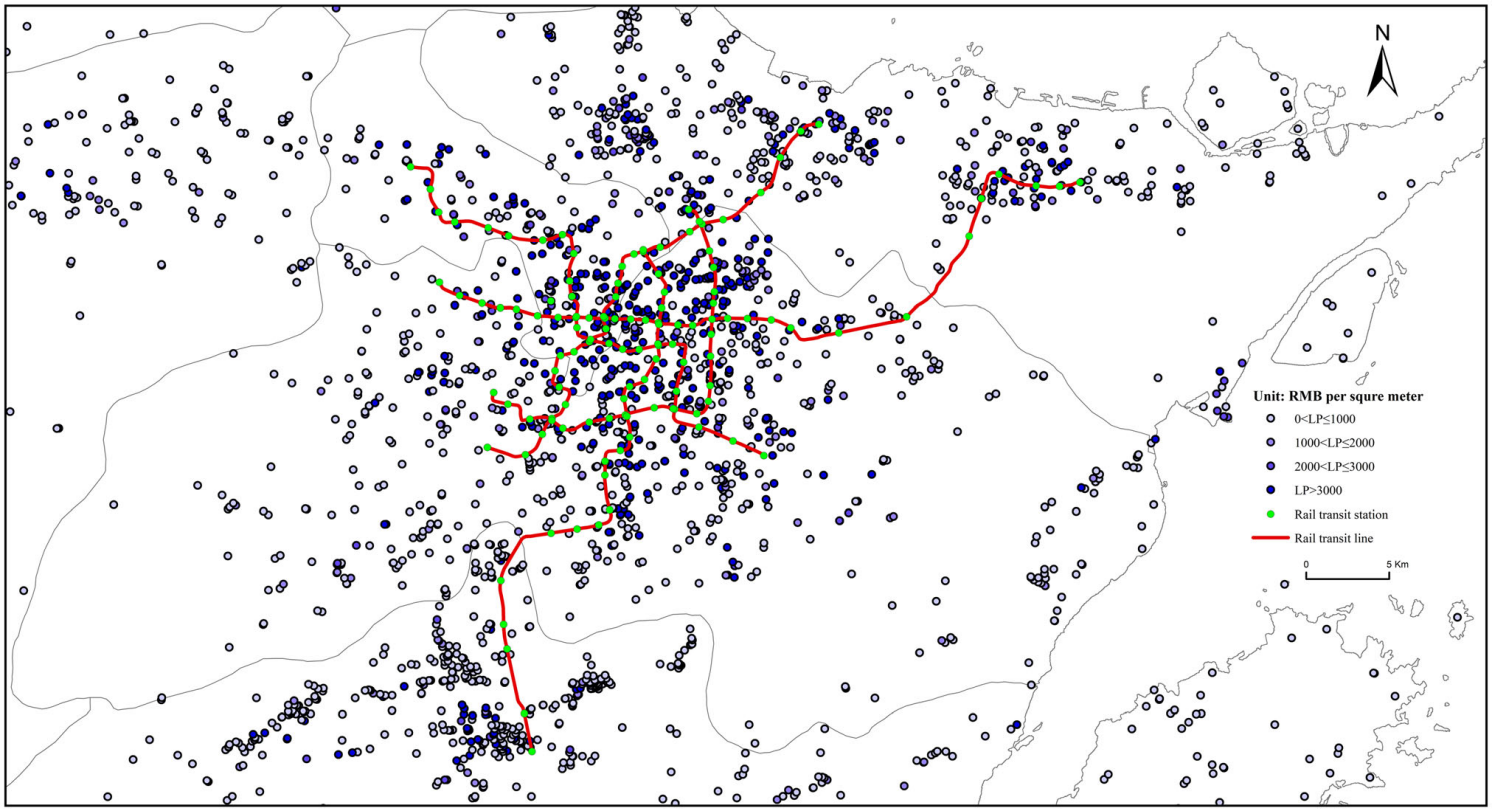
LTPs and URT system in Ningbo

Equation (1) presents the basic hedonic pricing model, indicating that LTP is constrained by the impacts of the land parcel’s distance to DisRTS, DisBTS, and DisHSR. Variables FAR and RTO were controlled to test the internal factor of the land parcels, as well as their impacts after RTO, respectively. Figure 3 shows a general trend, highlighting that the closer the land parcel is to the RTS, the higher the LTPs. Further, the DisPOI of public service was treated as a control variable, which can significantly reflect the impacts of the other factors—such as the other types of infrastructure development, land market cultivation and development, and urban vitality level—on the surrounding LTPs. To reduce the collinearity issue, the dependent variable, LTPs, is log-transformed, as expressed in Equation (2).

1 $$LTP \, = \, f\left( {DisRTS, \, DisBTS, \, DisPOI, \, DisHSR, \, FAR, \, RTO} \right)$$

2 $$lnLTP \, = \, f\left( {DisRTS, \, DisBTS, \, DisPOI, \, DisHSR, \, FAR, \, RTO} \right)$$

The hedonic pricing model revealed that Ningbo’s URT system exerts a significantly positive impact on LTPs (Table 2). Model 1 only examined the impact of DisRTS on LTPs, and the result demonstrates that LTP would decrease by ~5.6% for each 1-km increase in DisRTS. Model 2 controls the impact of DisPOI, and it still observed the significant land premium effects of URT in Ningbo at the 1% statistically significant level.

**Table 2:** Results of the hedonic pricing models

Variable	Model 1	Model 2	Model 3	Model 4	Model 5
DisRTS	−0.056*** (0.004)	−0.054*** (0.004)	−0.053*** (0.004)	−0.052*** (0.004)	−0.050*** (0.005)
DisBTS			−0.017 (0.026)		−0.024 (0.027)
DisHSR				−0.003 (0.002)	−0.003 (0.002)
DisPOI		−0.035 (0.052)	−0.014 (0.062)	−0.039 (0.052)	−0.009 (0.062)
FAR	0.601*** (0.016)	0.601*** (0.016)	0.601*** (0.016)	0.597*** (0.016)	0.597*** (0.016)
RTO	0.289*** (0.037)	0.288*** (0.037)	0.289*** (0.037)	0.289*** (0.037)	0.289*** (0.037)
Constant	5.854*** (0.035)	5.858*** (0.035)	5.855*** (0.035)	5.888*** (0.043)	5.889*** (0.043)
R-squared	0.275	0.276	0.276	0.276	0.276
F-value	638.42***	478.88***	383.14***	383.43***	319.65***
Obs.	5035	5035	5035	5035	5035

(1) Standard errors in parentheses; (2) ***p < 0.01, **p < 0.05, and *p < 0.1

Models 3 and 5 revealed that DisBTSs could also exert impacts on the surrounding LTPs. BTSs are much denser within core urbanized districts, significantly reflecting the role of bus transit proximity and those of other socioeconomic factors. Compared with regular bus transit, URT is more capital intensive. Thus, the impacts of bus transit are much lower and less statistically significant than that of URT. Generally, DisHSR stations exert an insignificant impact on LTPs (Model 4), probably because HSR stations are few and scattered around urbanized areas, mainly servicing intercity travel requirements. Model 6 examined the impacts of the DisURT station on LTPs after considering all the independent and controlled variables. FAR exerted positive and significant impacts in each model, reflecting the significant role of density and the internal factors of the land parcels in increasing LTPs. The RTO variable also reflects a significant land price increment after the URT system became operational. The coefficient of variable RTO revealed that the average LTP was 28.9% higher than that before the operation of URT in Ningbo. Model 6 reveals that LTP would decrease by ~5% for each 1-km increase in the DisURT station. The results obtained from these hedonic pricing models correlate with those of previous studies, but the land premium effect of URT was relatively lower than those reported in those studies [11, 12]. The land premium effects exhibit multiple helpful implications for local government TOD policies and practices.

## Policy Insights of the Land Premium Effects of URT for TOD Practice

Under China’s public land leasing regulations, it is vital to better implement LVC strategies and fully utilize the land premiums associated with the local contexts. Indeed, China’s local governments have considerably committed to exploiting the significant land-value increment owing to URT spillover effects to promote urban (re)development under different TOD policies and practices. Regarding the significant positive externalities of URT on land prices, Ningbo municipal government has issued several policies or guidelines to promote development-based LVC strategies, and these policies and guidelines would serve as a reference for promoting TOD and urban (re)development related to the local contexts.

### LVC as a Basis for Sustainable Rail Transit Investment

High-density and high-mixed land use represent an essential strategy in urban development to respond to the potential negative externality of rapid urbanization. When constructing the URT system, local governments are generally required to bear 40–50% of the total cost. The large-scale infrastructure investments for URT also generate considerable debt for such local governments. For example, at the end of 2021, the total debt of local governments was more than ¥30.47 trillion, which accounted for 26.64% of total GDP that year.

To reduce the financial burden and local debt risk in URT investments, the integrated development of rail transit and land use from an LVC perspective can be promoted, especially considering its significant positive impacts on LTPs. The Ningbo municipal government has issued several policies to promote URT construction and associated land development. Regarding URT financing and the investment policy, the municipal and district-level governments through which the rail transit line passes jointly bear the costs of land acquisition and rail transit construction. For comprehensive land development, Ningbo adopts the Rail + Property strategy within certain RTSs. This model can promote high-density and mixed land-use types, as well as enhance the connection between RTSs and land parcels, thus increasing travel convenience and accessibility, as well as ridership and fare-box revenue. Employing this TOD process, local rail transit companies can enjoy some land premiums, which can serve as an additional source for URT investments.

To promote the efficiency and effectiveness of the Rail + Property strategy, local governments must establish certain governing institutions to help local rail transit companies gain land development rights (LDR) through a market auction mechanism. The Ningbo municipal government actively promotes land reservation along with rail transit through certain policy innovations. In November 2013, Ningbo municipal government proposed the establishment of the “*One Database and Four Plans*” of land reservation for TOD practice to further strengthen land-reserve management and optimize the regulation of the land market. One database was a comprehensive set of land reservation and land supply conditions at the city level, serving as a foundation for the local government to formulate strategies for improved land reserve, land use, and land supply. The four proposed plans of the government included annual land acquisition and storage plans, preliminary land-development plans, land-supply plans, and land development capital plans. These innovative policies can better promote land-use planning and development in advance within the URT serving areas and grant potential LDRs to local rail transit companies, thereby promoting the sustainable development of URT through the development-based LVC.

### PPC as a Strategy for Amplifying Premium Effects

Since LDR must be transferred through a market auction mechanism, it is more competitive for local rail transit companies to gain LDRs by inviting a real estate developer with market competitiveness. Thus, such a PPC initiative can further promote the positive impacts of URT on land and housing prices. Yangliujun—a large TOD community in Ningbo—is such a typical case through the PPC initiative. Yangliujun is located in Ningbo’s Yinzhou District, close to the Qiuga East station of Ningbo rail transit Line 1. Yangliujun is positioned as a large-scale comprehensive community, which integrates land use for residential, commercial, and public services. By cooperating with the Greentown Real Estate Group—one of the famous real estate developers in China—through the primary and secondary development of land, the housing prices of the Yangliujun project are also growing rapidly.

In 2016, the Ningbo Rail Transit Group and Greentown Real Estate Group reached a cooperative development agreement. To identify a competent partner (land developers) for the co-development of the Yangliujun project, the Ningbo Rail Transit Group had set very strict requirements. For example, regarding corporate branding, the requirement is for a potential cooperative company to be among the top 20 real estate companies in China, and for their qualification regarding development, the participating companies were required to exhibit considerable experience in first-level land development. Additionally, the potential cooperative company was required to exhibit significant experience in the integrated development of land and RTSs, of which the development area of a single integrated project must have been more than 200,000 m^2^. After a series of complex negotiations, Ningbo Rail Transit Group and Greentown Real Estate Group contributed ¥100 million (49%) and ¥140.9 million (51%), respectively, to account for the development of the Yangliujun project.

In the Yangliujun project, LDRs were acquired through an open-bidding market mechanism. Through the PPC processes, the financial strength, service quality, planning concept, and market-forecasting ability of private enterprises became relevant factors for promoting joint Rail + Property development. The premium effect and potential LVC cannot be achieved without a mature and stable market condition, as well as close and effective cooperation with the private sector. Furthermore, this cooperation has exerted positive effects of PPC on premiums. The main duty of the rail transit group is to sustain rail transit construction and operation, and the introduction of land developers can greatly enhance the local rail transit groups’ experience in land development, as well as promote the land premium effect. For example, the Greentown Real Estate Group is an experienced developer of properties integrated with rail transit services, and this improves the quality of TOD development and operation, thereby increasing the premium feedback to the rail transit sector. Additionally, the PPC-supported Rail + Property development can exert a win–win effect. Regarding the Ningbo Rail Transit Group, the PPC initiative in Yangliujun has laid the foundation for undertaking integrated development on other land parcels. For the Greentown Real Estate Group, the PPC process has provided a new opportunity to participate in other TOD projects in Ningbo, either independently or in cooperation with the Ningbo Rail Transit Group.

### Transit-Oriented Urban Regeneration as a Strategy for Promoting TOD Practices

The intervention of rail transit can affect the urban spatial structure, offer impetus and new development opportunities for urban renewal and promote sustainable urban development. Notably, urban redevelopment based on rail transit represents an essential form of TOD practice. The land redevelopment around Wuxiang Station in Ningbo is also a typical case.

Located in the Yinzhou District of Ningbo, Wuxiang town is adjacent to Ningbo’s new central business district (CBD). Wuxiang station is also part of Ningbo Rail Transit Line 1. The nearby Lijiayang village is mainly for agricultural and construction land uses. Considering the new development opportunities offered by rail transit, with new-added transport capacities and land value effects, the land in Lijiayang village was expropriated and planned as a new TOD community, including various land-use types for business, school, and residential purposes. Considering the diversified and highly dense land development, future land and housing prices would also increase significantly. According to the land development plan, approximately 25.9 hectares of land were expropriated, and the amount of compensation was about ¥35 million. The resettlement of the villagers would be built adjacent to Lijiayang village, with a higher construction intensity. Owing to its proximity to Ningbo’s new CBD, the accessibility of Wuxiang town has greatly improved via rail transit service, which can accommodate the commuting needs of the residents. Since the housing price of Wuxiang town is much lower than those of the new CBD areas, it can attract some nonlocals with higher incomes to rent or live there, representing a typical form of transit-oriented urban gentrification.

In sum, the transit-oriented urban redevelopment and gentrification around Wuxiang station can avail a good experience for the TOD practice. First, the intervention of rail transit can attract multiparty investment for local development, promote the formation of different industries and reshape the existing land-use types. Diversified land-use types can improve the value-added effect accrued from new rail transit services [39]. Meanwhile, the improvement of real estate value by rail transit is more significant and stable in the combination with TOD [40]. High-density construction promotes the improvement of land-use efficiency and greatly improves land values. Second, transit-oriented urban renewal involves multiple stakeholders with different interests. Therefore, the interests of all parties must be considered in depth in the urban renewal supported by URT. Third, rail transit-oriented urban redevelopment can certainly attract individuals or groups with higher incomes. Rising housing and land prices, as well as the influx of high-income individuals, might induce the replacement of the original low-income class, resulting in urban gentrification. Therefore, the reasonable allocation of the premium brought by rail transit must be further clarified in practice. For example, the reasonable allocation of premiums enables some vulnerable groups of residents to promote housing affordability. Although public transport does not directly reduce housing affordability and gentrification [41], the impact of rail transport on gentrification is heterogeneous in different regions and cities [42]; it is still necessary to further clarify how to reasonably distribute the premium brought by rail transport in TOD practice.

## Conclusion and Discussion

Large investments, mainly from local government budgets, in the URT system would persist in urban China; thus, the integration of URT investments and land-use development is still of significance for facilitating transit priority development [43]. The URT system can generate significant spillover effects on the prices of the surrounding lands. Therefore, these land premium effects can be connected to the existing TOD policy and practice. This research, which employed Ningbo city as a case study, examined the premium effects of URT on land prices, after which it proposed three crucial policy insights to enhance the effectiveness and efficiency of TOD. It proposed that (1) local URT can generate significant land premium effects and that local governments could adopt different innovative policies to implement LVC; (2) the local rail transit authority can significantly amplify the premium effects through the PPC initiative, although it must still address the fair distribution of premiums across multiple stakeholders to satisfy their different interests; and (3) transit-oriented urban regeneration can significantly influence land prices and induce potential gentrification, which would be further addressed in the TOD policy and practice.

This study offered certain policy and planning implications for other regions or countries wherein large investments in the URT system would continue. First, it is quite crucial to better understand the impacts of URT on the surrounding land prices exhibiting temporal and spatial heterogeneity, and this is fundamental for implementing LVC instruments. Second, it becomes very important to relate these positive externalities to the local efforts in implementing the LVC strategies within local contexts and innovative policies. The Ningbo municipal government has adopted different policies to promote TOD and the associated urban (re)development, such as a series of policies and promotion of PPC for TOD practice. For example, for those local rail transit departments lacking land development experience, close cooperation with the private sector or other public authorities with multiple advantages can facilitate the implementation of transit-oriented (re)development and LVC.

The assessment of the land-price increment effect of URT and the implementation of the LVC strategies would be complex and context-based. This study was also associated with certain limitations. First, a variety of variables and models were considered in the extant literature. The premium effect of rail transit is affected by many factors. However, owing to the limited availability of data, only several factors of DisPOI were included in this study. Additionally, since the result of rail transit investment and land-price increments vary significantly, some models that deal with the issue of spatial autocorrelation would be required. However, this study only employed hedonic pricing models to test the impact of URT on land prices. Third, the implementation of the LVC strategies is complex, and a fair and effective distribution of land-price increments was certainly required. However, this study availed three types of policy insights, which were associated with land premiums, although an in-depth study on the development process, influential factors, and general effects of the practices are also necessary. To better address these concerns, a future study would benefit a lot from conducting (1) a comprehensive examination of the land-price increment of rail transit facilitated by refined models that fully consider the spatial–temporal heterogeneity; (2) a careful examination of the multiple stakeholders’ participation in TOD planning and investment, as well as the mechanism and effects of the LVC strategies; (3) a reflection on equitable TOD when the transit-oriented regeneration induces a significant gentrification effect.
